# Detection of the *in vitro* modulation of *Plasmodium falciparum* Arf1 by Sec7 and ArfGAP domains using a colorimetric plate-based assay

**DOI:** 10.1038/s41598-020-61101-3

**Published:** 2020-03-06

**Authors:** Tarryn Swart, Farrah D. Khan, Apelele Ntlantsana, Dustin Laming, Clinton G. L. Veale, Jude M. Przyborski, Adrienne L. Edkins, Heinrich C. Hoppe

**Affiliations:** 1 Department of Biochemistry & Microbiology, Rhodes University, Makhanda, 6140 South Africa; 2 School of Chemistry & Physics, Pietermaritzburg campus, University of KwaZulu-Natal, Private Bag X01, Scottsville, 3209 South Africa; 3 Center of Infectious Diseases, Parasitology, University of Heidelberg Medical School, INF324, 69210 Heidelberg, Germany; 4 Biomedical Biotechnology Research Unit (BioBRU), Rhodes University, Makhanda, 6140 South Africa; 5 Centre for Chemico- and Biomedicinal Research, Rhodes University, Makhanda, 6140 South Africa; 6 Present Address: Department of Molecular & Cell Biology, University of Cape Town, Rondebosch, 7700 South Africa

**Keywords:** Biochemistry, Biological techniques, Cell biology, Drug discovery

## Abstract

The regulation of human Arf1 GTPase activity by ArfGEFs that stimulate GDP/GTP exchange and ArfGAPs that mediate GTP hydrolysis has attracted attention for the discovery of Arf1 inhibitors as potential anti-cancer agents. The malaria parasite *Plasmodium falciparum* encodes a Sec7 domain-containing protein - presumably an ArfGEF - and two putative ArfGAPs, as well as an Arf1 homologue (*Pf*Arf1) that is essential for blood-stage parasite viability. However, ArfGEF and ArfGAP-mediated activation/deactivation of *Pf*Arf1 has not been demonstrated. In this study, we established an *in vitro* colorimetric microtiter plate-based assay to detect the activation status of truncated human and *P. falciparum* Arf1 and used it to demonstrate the activation of both proteins by the Sec7 domain of ARNO, their deactivation by the GAP domain of human ArfGAP1 and the inhibition of the respective reactions by the compounds SecinH3 and QS11. In addition, we found that the GAP domains of both *P. falciparum* ArfGAPs have activities equivalent to that of human ArfGAP1, but are insensitive to QS11. Library screening identified a novel inhibitor which selectively inhibits one of the *P. falciparum* GAP domains (IC_50_ 4.7 µM), suggesting that the assay format is suitable for screening compound collections for inhibitors of Arf1 regulatory proteins.

## Introduction

ADP-ribosylation factor (Arf) GTPases are central regulators of protein trafficking in eukaryotic cells. There are six Arf isoforms, divided into three classes based on sequence homology, of which the most widely studied are Arf1 (Class I) and Arf6 (Class III). Respectively, they principally mediate trafficking in the secretory (Arf1) and endocytic (Arf6) pathways, with additional roles for Arf6 in actin cytoskeleton dynamics^1–3^. Arf1 is the focus of this study and initiates vesicle formation in the Golgi apparatus by activating lipid modifying enzymes and recruiting coatomer complex I (COPI) coat proteins. The COPI vesicles are responsible for retrograde transport of cargo and trafficking proteins to earlier Golgi compartments and the endoplasmic reticulum^4^. In addition, Arf1 recruits clathrin adaptor proteins (AP1, AP3 and AP4) and Golgi-localized γ-ear-containing ARF-binding (GGA) proteins to the *trans*-Golgi network, where they are involved in trapping cargo proteins and the formation of vesicles that deliver secretory proteins to endosomes^5^.

Presumably, the delivery of newly synthesised secretory proteins to their correct locations places a heavy burden on Arf1 activity in rapidly growing cells. Indeed, Arf1 is upregulated in cancer cell types and plays a role in cancer metastasis phenotypes e.g. cell detachment, migration and invasion, and may additionally be involved in tumour-promoting cell signalling pathways e.g. the phosphatidylinositol 3-kinase (PI3K) and mitogen-activate protein kinase (MAPK) pathways^6–9^. Moreover, Arf1 inhibitors inhibit cancer cell viability, proliferation and metastatic characteristics^10^ and tumour growth in mouse models^11–13^. Like other small GTPases, Arf1 undergoes a cycle of activation and deactivation that is determined by its nucleotide binding status. Exchanging GDP for GTP activates Arf1 through a pronounced conformational change which exposes a myristoylated N-terminal amphipathic α-helix, resulting in membrane association, and enhances effector protein binding. Conversely, hydrolysis of the terminal phosphate of the bound GTP to form GDP deactivates Arf1, returning it to a cytoplasmic pool. Due to the low intrinsic nucleotide exchange and hydrolysis activity of Arf1, Arf1 activation is stimulated by a family of guanine nucleotide exchange factors (GEFs) containing a characteristic Sec7 domain^14^, while deactivation is promoted by GTPase activating proteins (GAPs) containing GAP domains^14,15^. The development of Arf1 inhibitors has focused on compounds that disrupt GEF-mediated nucleotide exchange (although the detailed mechanisms may differ) and includes *inter alia* brefeldin A (BFA) and its analogues, Golgicide A, AMF-26, LM11, Exo2 and SecinH3^11,16–20^. However, Arf GAP inhibitors – QS11 and its derivatives – have been described and reported to inhibit the migration of breast cancer cells^21,22^.

The genome of the most prevalent and virulent of the malaria parasite species, *Plasmodium falciparum*, contains six sequences that have been annotated as encoding putative Arf or Arf-like proteins (www.plasmodb.org). One such sequence encodes an Arf1 homologue (*Pf*Arf1) that has a very high amino acid sequence conservation (76% identity, 89% similarity) compared to human Arf1. Originally identified by probing a *P. falciparum* genomic library and PCR from *P. falciparum* cDNA^23–25^, the recombinant protein was shown to bind GTP, have ADP-ribosyltransferase and phospholipase D stimulating activity in addition to low intrinsic GTPase activity, all features of Arf GTPases^24,25^. It is also capable of stimulating *P. falciparum* phosphatidylinositol 4-phosphate 5-kinase (PIP5K), which is an established role of mammalian Arf1 in the regulation of phosphorylated phosphatidylinositol levels and, consequently, membrane trafficking, signalling and cytoskeleton dynamics^26^. In blood-stage parasites, *Pf*Arf1 fused to GFP was found to co-localise with the Golgi marker GRASP^27^, while the canonical Arf1 activation inhibitor BFA causes a disruption in Golgi architecture and trafficking of secretory proteins^28–32^. Taken together, these studies suggest that *Pf*Arf1 mimics the key role of mammalian Arf1 in secretory traffic through the Golgi apparatus. As would be expected based on sequence conservation, the crystal structure of GDP-bound *Pf*Arf1is very similar to that of human Arf1, with subtle differences in the Switch I and II domains that could affect binding of GEFs and GAPs^33^. However, direct demonstration of GEF-mediated nucleotide exchange and GAP-mediated GTP hydrolysis by *Pf*Arf1 has not been reported.

Interestingly, unlike mammalian cells where the Arf GEF and GAP families contain up to 15 and 27 members respectively^14^, the *P. falciparum* genome encodes two putative ArfGAP proteins and a single Sec7 domain-containing putative ArfGEF, responsible for the BFA sensitivity of malaria parasites^34,35^. The crystal structure of the catalytic GAP domain of one of the GAP isoforms (designated *Pf*ArfGAP1 in this study) has been determined and shows an overall similarity of tertiary structure compared to mammalian GAP domains^36^. However, unlike the highly conserved *Pf*Arf1, there is a greater divergence of amino acid sequence homology compared to human ArfGAP1 (39% identity and 52% similarity) and differences in the amino acid residues predicted to interact with Arf1^36^. In this study, using human recombinant proteins as a model, we developed a novel microtiter plate-based assay to detect Arf1 activation (GTP vs. GDP-bound) status and modulation of it by an ArfGEF (ARNO) Sec7 domain and Arf GAP (ArfGAP1) GAP domain. We used the assay to demonstrate and compare the Arf1 GAP activities of the GAP domains of the two putative *P. falciparum* GAPs, as well as demonstrate ARNO-stimulated nucleotide exchange by *Pf*Arf1. Given the interest in Arf1 as a drug target, a further motivation for developing the assay was to introduce an assay format compatible with the screening of compound libraries for Arf1 activity modulators, explored here by detecting the differential inhibition of ARNO and GAP-mediated Arf1 activation/deactivation using standard inhibitors, as well as the identification of a novel, selective *Pf*Arf1 GAP inhibitor.

## Results

### A colorimetric plate-based GST-GGA3 binding assay discriminates between GTP- and GDP-bound Arf1

**Figure 1:**
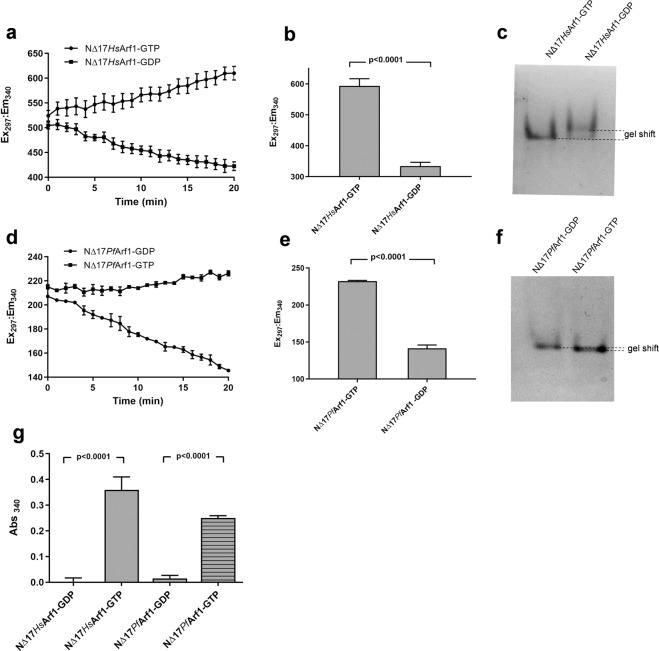
Microtiter plate GST-GGA3^GAT^ binding assay using GTP and GDP preloaded Arf1 proteins. (**a**,**d**) Five µM ^NΔ17^*Hs*Arf1 (**a**) and ^NΔ17^*Pf*Arf1 (**d**) were incubated at 25 °C with 50 µM GTP or GDP in the presence of 2 mM (^NΔ17^*Hs*Arf1) or 20 mM (^NΔ17^*Pf*Arf1) EDTA in a black 96-well plate and tryptophan fluorescence (Ex_297_/Em_340_) measured at 1 min intervals in a plate reader for 20 min. (**b**,**e**) After a further 40 min incubation, MgCl_2_ was added to a final concentration of 3 mM (^NΔ17^*Hs*Arf1) or 30 mM (^NΔ17^*Pf*Arf1), incubation continued for 10 min and ^NΔ17^*Hs*Arf1 (**b**) and ^NΔ17^*Pf*Arf1 (**e**) tryptophan fluorescence measured as an end-point reading. The nucleotide exchange reactions were conducted in triplicate wells and the data points represent mean fluorescence ± standard deviation. (**c**,**f**) After completion of nucleotide exchange, GTP and GDP loaded ^NΔ17^*Hs*Arf1 (**c**) and ^NΔ17^*Pf*Arf1(**f**) were run on 12% native PAGE gels and stained with Coomassie. The gel images were cropped from two separate native PAGE gels, shown in Fig. S6 (Supporting Information). (**g**) GTP and GDP preloaded ^NΔ17^*Hs*Arf1 and ^NΔ17^*Pf*Arf1were added to the wells of a Ni-NTA coated clear 96-well plate at a concentration of 1 µM and incubated for 30 min at 4 °C. An equal volume of GST-GGA3^GAT^ was added to a final concentration of 1 µM and incubation continued for 60 min. After washing the wells, GST substrate solution containing reduced L-glutathione and 1-chloro-2,4-dinitrobenzene was added and absorbance measured at 340 nm after a 30 min incubation at room temperature. Mean background absorbance values obtained from empty wells (i.e. lacking immobilised Arf1) incubated with GST-GGA3^GAT^ followed by GST substrate were subtracted from experimental readings. Incubations were carried out in triplicate wells and the bars represent mean Abs_340_ ± standard deviation. P-values were calculated by two-tailed t-tests.

The phenomenon that Arf1only binds to the coat protein GGA3 (via the GAT domain of the latter) when it is in its active GTP-bound vs. inactive GDP-bound conformation has been widely employed as an experimental tool to detect Arf1 activation status in cultured cells using pull-down assays. Typically, glutathione beads coated with a fusion protein consisting of glutathione-S-transferase (GST) and the GAT domain of GGA3 (GST-GGA3^GAT^) are incubated with cell lysates and bead-bound (active) vs. total Arf1 levels determined by western blotting^37^. To determine if the selective binding of GST-GGA3^GAT^ to Arf1-GTP could be further exploited to determine the activation status of purified recombinant Arf1 proteins in a microtiter plate format, we conceptualised an assay procedure (Fig. S1) in which Arf1, expressed and purified as a truncated histidine-tagged protein (Fig. S2), is immobilised on nickel-NTA coated 96-well plates, followed by incubation with purified GST-GGA3^GAT^. The extent of GST-GGA3^GAT^ binding to the plate may be readily determined by the addition of a colorimetric GST enzyme substrate, and should correlate with the level of GTP-bound Arf1. Assessing the viability of this approach required the preparation of GTP-bound and GDP-bound Arf1, respectively, which was achieved by a standard method^38^. His-tagged human and *P. falciparum* Arf1, minus the N-terminal 17 amino acids containing the myristoylation site and amphipathic α-helix (^NΔ17^*Hs*Arf1and ^NΔ17^*Pf*Arf1, respectively), were incubated with GTP or GDP in the presence of EDTA, followed by the addition of Mg^2+^ to stabilise the attached nucleotide. The *Hs*Arf1 conformational change induced by GTP binding was monitored by kinetic and end-point intrinsic tryptophan fluorescence reads (Fig. 1a,b), as well as by performing native PAGE on the final protein preparations (Fig. 1c). As anticipated by the high level of sequence conservation in *Pf*Arf1, the results confirmed that this is a viable approach for preparing and assessing GTP- and GDP-bound ^NΔ17^*Pf*Arf1 (Fig. 1d–f), although the native PAGE mobility difference between ^NΔ17^*Pf*Arf1-GTP and -GDP was smaller than observed with the human protein. The kinetic tryptophan fluorescence measurements further suggested that the original ^NΔ17^*Hs*Arf1preparation was purified from *E. coli* as a mixture of GDP- and GTP-bound proteins (based on the respective increase and decrease in fluorescence during incubation with GTP and GDP), while ^NΔ17^*Pf*Arf1 was predominantly GTP-bound.

To determine if GST-GGA3^GAT^ could be used to detect Arf1 activation status using the plate-based colorimetric assay format described above, the nucleotide-loaded Arf1 proteins were incubated in a nickel-NTA coated 96-well plate, followed by sequential incubations with GST-GGA3^GAT^ and a colorimetric GST substrate and absorbance readings performed at 340 nm (Fig. 1g). GTP- vs GDP-bound ^NΔ17^*Hs*Arf1could be robustly distinguished by the level of GST enzyme activity captured in the wells and the ^NΔ17^*Pf*Arf1 results further confirmed that selective nucleotide-dependent GGA3^GAT^ binding ability is conserved in the malaria protein. To confirm that selective binding of ^NΔ17^*Pf*Arf1-GTP to GST-GGA3^GAT^ was due to the recognition of the GGA3^GAT^ portion of the fusion protein, we found that untagged GST failed to bind to ^NΔ17^*Pf*Arf1-GTP (or -GDP) (Fig. S3). In addition, GTP- vs. GDP-bound ^NΔ17^*Pf*Arf1 was preferentially co-precipitated by GGA3-coated beads (Fig. S3).

### Detection of ARNO-mediated nucleotide exchange by human and P. falciparum Arf1

**Figure 2:**
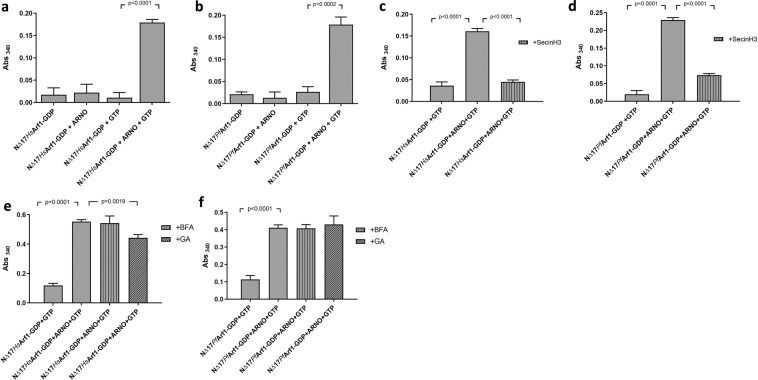
Detection of ARNO-mediated nucleotide exchange using the GST-GGA^GAT^ binding assay. (**a**,**b**) One µM GDP preloaded ^NΔ17^*Hs*Arf1 (**a**) or ^NΔ17^*Pf*Arf1 (**b)** was incubated with 0.2 µM ARNO^Sec7^ and 50 µM GTP for 30 min at 37 °C, added to Ni-NTA coated 96-well plates and incubated for a further 30 min at 4 °C. GST-GGA3^GAT^ was added to 1 µM and incubation continued at 4 °C for 60 min, followed by washing, incubation with GST substrate and absorbance readings at 340 nm. Control incubations contained the respective GDP preloaded Arf1 proteins alone, GDP preloaded Arf1 incubated with ARNO^Sec7^ in the absence of GTP and GDP preloaded Arf1 incubated with GTP in the absence of ARNO^Sec7^. (**c–f**) ARNO^Sec7^ nucleotide exchange reactions were repeated with GDP preloaded ^NΔ17^*Hs*Arf1 and ^NΔ17^*Pf*Arf1in the presence of 50 µM SecinH3 (**c,d**) Brefeldin A (BFA) or Golgicide A (GA) (**e,f**), followed by the GST-GGA3^GAT^ binding assay. Control reactions consisted of the GDP preloaded Arf1 proteins incubated with GTP in the absence of ARNO^Sec7^ and inhibitors. Mean Abs_340_ values obtained from empty Ni-NTA plate wells incubated with GST-GGA3^GAT^ were subtracted from all other readings. Incubations were carried out in triplicate wells and Abs_340_ shown as mean ± standard deviation. P-values were derived from two-tailed t-tests.

To determine if the assay can be further exploited to detect the activation of Arf1 by an ArfGEF *in vitro*, GDP-loaded ^NΔ17^*Hs*Arf1 and ^NΔ17^*Pf*Arf1 were incubated with GTP in the presence of the Sec7 domain of ARNO (ARNO^Sec7^) before adding the reactions to nickel-NTA coated plates and proceeding with the assay described above. ARNO^Sec7^-mediated nucleotide exchange by both ^NΔ17^*Hs*Arf1 and ^NΔ17^*Pf*Arf1 could be discerned by a marked increase in GST-GGA3^GAT^ binding compared to the respective controls (Fig. 2a,b). The controls consisted of the GDP-bound Arf1 proteins (^NΔ17^*Hs*Arf1-GDP and ^NΔ17^*Pf*Arf1-GDP), the GDP-bound Arf1 proteins incubated with ARNO^Sec7^ in the absence of GTP, and the GDP-bound Arf1 proteins incubated with GTP in the absence of ARNO^Sec7^. To confirm that the enhanced GST-GGA3^GAT^ binding was due to an increase in Arf1-GTP levels caused by ARNO^Sec7^ stimulated nucleotide exchange, the reactions were repeated in the presence of 50 µM SecinH3, an inhibitor of the cytohesin family of ArfGEFs to which ARNO belongs^20^. Inclusion of SecinH3 in the ARNO^Sec7^ exchange reaction reduced GST-GGA3^GAT^ binding by both ^NΔ17^*Hs*Arf1 and ^NΔ17^*Pf*Arf1 to levels obtained with control reactions lacking ARNO^Sec7^ (Fig. 2c,d), causing a 93% and 74% inhibition of ARNO^Sec7^-mediated ^NΔ17^*Hs*Arf1 and ^NΔ17^*Pf*Arf1 nucleotide exchange, respectively. The exchange reactions were subsequently repeated in the presence of 50 µM brefeldin A (BFA) or Golgicide A (GA), which are more selective for the BIG and GBF families of ArfGEFs as opposed to cytohesins^17,38^. Consistent with this bias, neither compound inhibited ARNO^Sec7^-mediated ^NΔ17^*Pf*Arf1 activation (Fig. 2f), while Golgicide A caused only a minor 26% inhibition of ^NΔ17^*Hs*Arf1 nucleotide exchange (Fig. 2e). In summary, the results confirmed that *Pf*Arf1 is susceptible to Sec7-mediated nucleotide exchange *in vitro*. In addition, it suggested that the assay format can robustly detect the *in vitro* activation Arf1 by a Sec7 domain, as well as the specific inhibition of the reaction by small compound inhibitors.

### Detection of GAP-mediated GTP hydrolysis by human and *P. falciparum* Arf1

**Figure 3:**
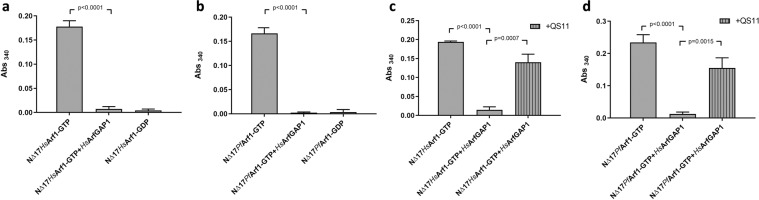
Detection of GAP-mediated Arf1 deactivation using the GST-GGA^GAT^ binding assay. (**a,b**) One µM GTP preloaded ^NΔ17^*Hs*Arf1 (**a**) or ^NΔ17^*Pf*Arf1 (**b**) was incubated with 0.1 µM *Hs*ArfGAP1^GAP^ for 30 min at 37 °C, transferred to a Ni-NTA coated 96-well plate and incubation continued at 4 °C for 30 min. GST-GGA3^GAT^ was added to 1 µM and incubation at 4 °C continued for 60 min, followed by washing, incubation with GST substrate and absorbance readings at 340 nm. Control reactions consisted of GTP preloaded Arf1 proteins incubated in the absence of *Hs*ArfGAP1^GAP^ and wells incubated with GDP preloaded Arf1 proteins alone. (**c**,**d**) The incubations of ^NΔ17^*Hs*Arf1 (**c**) and ^NΔ17^*Pf*Arf1 (**d**) with *Hs*ArfGAP1^GAP^ were repeated in the presence of 50 µM QS11. Control reactions consisted of incubations of the GTP preloaded Arf1 proteins in the absence of *Hs*ArfGAP1^GAP^ and QS11. Abs_340_ values obtained from empty Ni-NTA plate wells incubated with GST-GGA3^GAT^ were subtracted from all other readings. Incubations were carried out in triplicate wells and Abs_340_ shown as mean ± standard deviation. P-values were calculated by two-tailed t-tests.

Having demonstrated *in vitro* Sec7-mediated nucleotide exchange by *Pf*Arf1, we next explored whether the assay format could detect GAP-mediated *Pf*Arf1 deactivation, using the GAP domain of human ArfGAP1 (*Hs*ArfGAP1^GAP^) as a model GAP. ^NΔ17^*Hs*Arf1 and ^NΔ17^*Pf*Arf1 preloaded with GTP were incubated with *Hs*ArfGAP1^GAP^, added to a nickel-NTA plate and GST-GGA3^GAT^ binding assessed (Fig. 3a,b). Controls included the GTP-loaded Arf1 proteins incubated in the absence of *Hs*ArfGAP1^GAP^ and plate wells containing immobilised GDP-loaded Arf1 proteins. Incubation with the GAP domain completely abrogated the binding of GST-GGA3^GAT^ to both ^NΔ17^*Hs*Arf1 and ^NΔ17^*Pf*Arf1. To confirm that this was due to GAP-stimulated inactivation (GTP hydrolysis) of the Arf1 proteins, the ArfGAP inhibitor QS11^21^ was included in the incubations of the GTP-loaded Arf1 proteins with *Hs*ArfGAP1^GAP^ at a concentration of 50 µM, which preserved GST-GGA3^GAT^ binding of both ^NΔ17^*Hs*Arf1 and ^NΔ17^*Pf*Arf1 (Fig. 3c,d). Collectively, the results confirmed that *Pf*Arf1 is susceptible to GAP-mediated deactivation and that the assay format can competently detect *in vitro* ArfGAP activity as well as its inhibition by a small molecule inhibitor.

### GAP activity of two putative P. falciparum ArfGAPs

**Figure 4:**
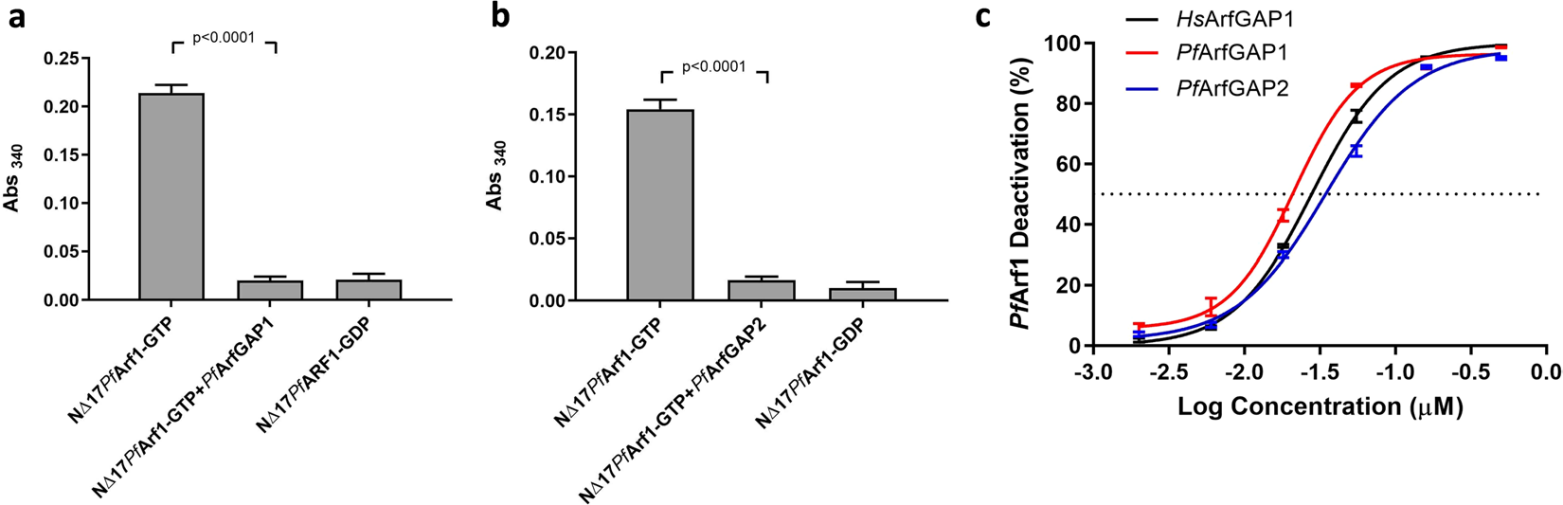
Detection of GAP activity of *P. falciparum* GAP domains using the GST-GGA^GAT^ binding assay. (**a**,**b**) One µM GTP preloaded ^NΔ17^*Pf*Arf1 was incubated with 0.1 µM *Pf*ArfGAP1^GAP^ (**a**) or *Pf*ArfGAP2^GAP^ (**b**) for 30 min at 37 °C, transferred to a Ni-NTA coated 96-well plate and incubation continued at 4 °C for 30 min. GST-GGA3^GAT^ was added to 1 µM and incubation at 4 °C continued for 60 min, followed by washing, incubation with GST substrate and absorbance readings at 340 nm. Control reactions consisted of GTP preloaded ^NΔ17^*Pf*Arf1 incubated in the absence of the respective GAP domains and wells incubated with GDP preloaded ^NΔ17^*Pf*Arf1 alone. Abs_340_ values obtained from empty Ni-NTA plate wells incubated with GST-GGA3^GAT^ were subtracted from all other readings. Incubations were carried out in triplicate wells and Abs_340_ is shown as mean ± standard deviation. P-values were calculated using two-tailed t-tests. (**c**) One µM GTP preloaded ^NΔ17^*Pf*Arf1 was incubated with three-fold serial dilutions (0.5–0.002 µM) of *Pf*ArfGAP1^GAP^, *Pf*ArfGAP2^GAP^ and *Hs*ArfGAP1^GAP^ for 30 min at 37 °C and the GST-GGA3^GAT^ binding assay carried out as described above. Percentage *Pf*Arf1 deactivation was calculated from the Abs_340_ values obtained at the various GAP domain concentrations relative to those obtained with ^NΔ17^*Pf*Arf1-GTP (0%) and ^NΔ17^*Pf*Arf1-GDP (100%) incubated in the absence of GAP domains. Dose-response curves of percentage *Pf*Arf1 deactivation vs. Log[GAP concentration] were generated by non-linear regression analysis using GraphPad Prism.

To some extent, stimulation of *Pf*Arf1 nucleotide exchange and GTP hydrolysis by human Sec7 and GAP domains (as well as GTP-dependent binding to the human effector protein GGA3) was not unexpected, given the high sequence and structural conservation of *Pf*Arf1^33^. However, the question remains to what extent the predicted endogenous *P. falciparum* GEF and GAPs are capable of acting on *Pf*Arf1. In this study, we focused on the two sequences which are annotated as ArfGAPs on the plasmodb.org malaria genome database, which we designated as *Pf*ArfGAP1 (Plasmodb entry PF3D7_1244600) and *Pf*ArfGAP2 (PF3D7_0526200.1). In contrast to the sequence conservation of *Pf*Arf1, the predicted amino acid sequences of the GAP domains of two proteins are considerably less conserved compared to human ArfGAPs (alignments with *Hs*ArfGAP1 given in Supplementary Information Fig. S3) and, while the crystal structure of the *Pf*ArfGAP1 GAP domain has been published^36^, neither GAP domain has been reported to have catalytic activity. To demonstrate the latter, we repeated the assays performed with *Hs*ArfGAP1^GAP^. GTP-loaded ^NΔ17^*Pf*Arf1 was incubated with the GAP domains of the respective malarial ArfGAPs (*Pf*ArfGAP1^GAP^, *Pf*ArfGAP2^GAP^) and GST-GGA3^GAT^ binding assessed (Fig. 4a,b). As was previously found with *Hs*ArfGAP1^GAP^, both GAP domains reduced GST-GGA3^GAT^ binding to the levels obtained with the GDP-loaded ^NΔ17^*Pf*Arf1 controls, suggesting that they had stimulated GTP hydrolysis by the *Pf*Arf1 protein. As an end-point assay, the assay format employed here prevented a direct comparison of the GAP activity of the two GAP domains using a kinetic read-out of GTP hydrolysis by ^NΔ17^*Pf*Arf1. To address this, a GAP titration assay was performed. ^NΔ17^*Pf*Arf1-GTP was incubated at a concentration of 1 µM with serial dilutions of *Pf*ArfGAP1^GAP^, *Pf*ArfGAP2^GAP^ and *Hs*ArfGAP1^GAP^, GST-GGA3^GAT^ binding was determined and the dose-response curves compared (Fig. 4c). In this assay format, the GAP activities of the respective GAP domains were not found to be markedly different. Of the two *P. falciparum* GAP domains, *Pf*ArfGAP1^GAP^ was more active, with half-maximal GAP activity (EC_50_) at 0.021 µM, compared to 0.034 µM for *Pf*ArfGAP2^GAP^ (*Hs*ArfGAP1^GAP^ was intermediate at 0.028 µM).

### Identification of a selective small molecule inhibitor of PfArfGAP1^GAP^ activity

**Figure 5:**
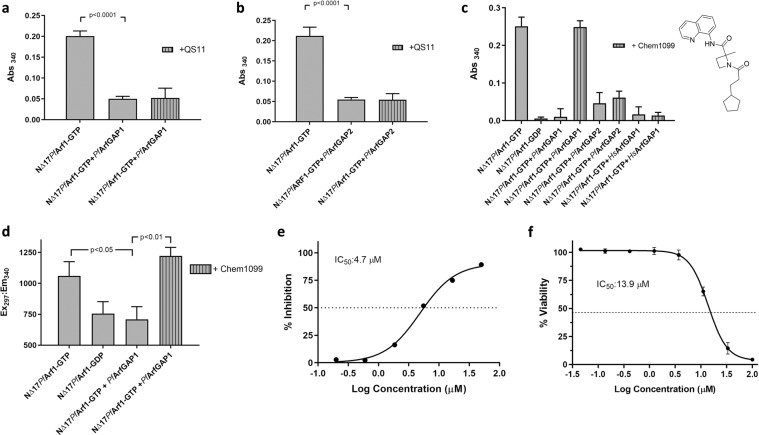
Selective inhibition of *Pf*ArfGAP1^GAP^ activity by a small molecule inhibitor. (**a**,**b**) One µM GTP preloaded ^NΔ17^*Pf*Arf1 was incubated with 0.1 µM *Pf*ArfGAP1^GAP^ (**a**) or *Pf*ArfGAP2^GAP^ (**b**) for 30 min at 37 °C in the presence of 50 µM QS11, transferred to a Ni-NTA coated 96-well plate and incubation continued at 4 °C for 30 min. GST-GGA3^GAT^ was added to 1 µM and incubation at 4 °C continued for 60 min, followed by washing, incubation with GST substrate and absorbance readings at 340 nm. Control reactions consisted of GTP preloaded ^NΔ17^*Pf*Arf1 incubated in the absence of the respective GAP domains, or with the GAP domains in the absence of QS11. Abs_340_ values obtained from empty Ni-NTA plate wells incubated with GST-GGA3^GAT^ were subtracted from all other readings. Incubations were carried out in triplicate wells and Abs_340_ is shown as mean ± standard deviation. (**c**) One µM GTP preloaded ^NΔ17^*Pf*Arf1 was incubated respectively with 0.1 µM *Pf*ArfGAP1^GAP^, *Pf*ArfGAP2^GAP^ or *Hs*ArfGAP1^GAP^ in the absence or presence of 50 µM Chem1099 and the GST-GGA3^GAT^ binding assay repeated as described above. Bars represent mean Abs_340 ± _standard deviation obtained from triplicate wells. The structure of Chem1099 is shown to the right. (**d**) Incubation of 1 µM ^NΔ17^*Pf*Arf1-GTP with 0.1 µM *Pf*ArfGAP1^GAP^ in the presence and absence of 50 µM Chem1099 for 30 min at 37 °C was repeated in a black 96-well plate and tryptophan fluorescence (Ex_297_/Em_340_) measured as an end-point reading. Additional wells contained 1 µM ^NΔ17^*Pf*Arf1-GDP without *Pf*ArfGAP1^GAP^ or without Chem1099. Bars represent mean fluorescence ± standard deviation obtained from triplicate wells. P-values were calculated by two-tailed t-tests. (**e**) The GST-GGA3^GAT^ binding assay with Chem1099 was repeated with three-fold serial dilutions (50 µM – 0.2 µM) of the compound added to the incubation of ^NΔ17^*Pf*Arf1-GTP with *Pf*ArfGAP1^GAP^ in triplicate wells. Percentage inhibition of *Pf*ArfGAP1^GAP^ activity was calculated from the Abs_340_ readings obtained at the various compound concentrations relative to the mean Abs_340_ obtained with ^NΔ17^*Pf*Arf1-GTP incubated with *Pf*ArfGAP1^GAP^ in the absence of Chem1099 (0%) and wells incubated with ^NΔ17^*Pf*Arf1-GTP alone (100%). A dose-response curve was generated from the plot of mean percentage *Pf*ArfGAP1^GAP^ inhibition ± standard deviation vs. Log(Chem1099 concentration) and the IC_50_ value derived by non-linear regression analysis using GraphPad Prism. (**f**) The antiplasmodial activity of Chem1099 was assessed by incubating *P. falciparum* (3D7) parasites with a serial dilution of Chem1099 in triplicate wells for 48 h and determining percentage parasite viability (relative to untreated controls) using a plasmodial lactate dehydrogenase assay. The IC_50_ value was derived by non-linear regression analysis of the % parasite viability vs. Log(Chem1099 concentration) plot using GraphPad Prism.

To confirm that the reduction in GST-GGA3^GAT^ binding when GTP preloaded ^NΔ17^*Pf*Arf1was incubated with 0.1 µM *Pf*ArfGAP1^GAP^ and *Pf*ArfGAP2^GAP^ was due to GAP activity, the assays were repeated in the presence of 50 µM QS11. In contrast to the results obtained with *Hs*ArfGAP1^GAP^ (Fig. 3d), QS11 was unable to restore GST-GGA3^GAT^ binding by ^NΔ17^*Pf*Arf1-GTP incubated with either *Pf*ArfGAP1^GAP^ or *Pf*ArfGAP2^GAP^ (Fig. 5a,b). To identify a potential inhibitor of *Pf*ArfGAP1^GAP^-mediated deactivation of ^NΔ17^*Pf*Arf1-GTP, we therefore screened a small BioFocus library of 1120 α-helix mimetics at a concentration of 50 µM (Screening details in Supplementary Information Fig. S5). We focused on the GAP domain of *Pf*ArfGAP1 since, in contrast to *Pf*ArfGAP2, the coding sequence has been reported to be essential to the survival of blood stage *P. falciparum* and *P. berghei* (murine malaria) parasites in genome-wide knockout and transposon mutagenesis studies^39,40^. This led to the identification of Chem1099 (Fig. 5c) which, at a concentration of 50 µM, preserved the GST-GGA3^GAT^ binding ability of ^NΔ17^*Pf*Arf1-GTP incubated with *Pf*ArfGAP1^GAP^, presumably due to inhibition of the GAP activity of the latter (Fig. 5c). Interestingly, the compound was inactive in a parallel screen carried out with *Pf*Arf1 and *Hs*ArfGAP1^GAP^ (not shown). Indeed, at 50 µM, Chem1099 failed to inhibit the GAP activity of either *Hs*ArfGAP1^GAP^ or *Pf*ArfGAP2^GAP^ on ^NΔ17^*Pf*Arf1-GTP, suggesting GAP selectivity (Fig. 5c). The inhibitory activity of Chem1099 was further confirmed using an alternative assay format. As described earlier, tryptophan fluorescence measurements can be used to assess the conformation of ^NΔ17^*Pf*Arf1 which reflects its GTP- vs. GDP-bound status. Incubation of ^NΔ17^*Pf*Arf1-GTP with *Pf*ArfGAP1^GAP^ reduced its tryptophan fluorescence to levels obtained with a ^NΔ17^*Pf*Arf1-GDP control, reflecting stimulation of GTP hydrolysis by the GAP domain (Fig. 5d). By contrast, inclusion of 50 µM Chem1099 in the reaction maintained ^NΔ17^*Pf*Arf1-GTP fluorescence levels, suggesting complete inhibition of *Pf*ArfGAP1^GAP^ GAP activity. Dose-dependent inhibition of *Pf*ArfGAP1^GAP^ activity by Chem1099 was demonstrated by incubating ^NΔ17^*Pf*Arf1-GTP and the GAP domain with serial dilutions of the compound followed by the GST-GGA3^GAT^ binding assay and yielded an IC_50_ value of 4.7 µM (Fig. 5e). To determine if Chem1099 possesses anti-parasitic activity, a dose-response assay was conducted against cultured *P. falciparum* (3D7) parasites and parasite viability assessed using a plasmodial lactate dehydrogenase assay, which yielded an IC_50_ of 13.9 µM (Fig. 5f). In conclusion, the results suggest that *Pf*ArfGAP1 GAP activity can be inhibited by small compounds *in vitro*, that inhibitory compounds can discriminate between the GAP domains used in this study and that the assay format can be used to identify GAP inhibitors in compound libraries.

## Discussion

Given the rapid growth rate of the *P. falciparum* malaria parasite and its reliance on vesicular trafficking to secrete proteins to internal organelles (notably specialised secretory organelles required for erythrocyte invasion), trafficking of proteins to and in the host erythrocyte cytoplasm, as well as extensive endocytosis of erythrocyte cytoplasm^41^, it is intriguing that, in contrast to mammalian cells, its genome only encodes one predicted Sec7 domain protein (ArfGEF) and two ArfGAPs (according to plasmodb.org annotations) to potentially regulate Arf GTPase function which is central to trafficking in mammalian cells. This is further compounded by the complexity of the parasite life-cycle which, in addition to the blood stages responsible for malaria pathogenesis, includes male and female gametocyte transmission stages, several stages in the *Anopheles* mosquito vector and human liver stages^42^. Moreover, although 6 sequences have been annotated as putative ADP-ribosylation factors, four may be Arf-like proteins as opposed to canonical Arf GTPases, one (Plasmodb accession number PF3D7_1034700) appears non-essential for blood-stage parasite survival^39,40^, and only *Pf*Arf1 has been characterised^23–27,33^. We have focused on *Pf*Arf1 and found that it binds to the GAT domain of the human effector protein GGA3 in a nucleotide-dependent manner, which allows it to be characterised *in vitro* using the plate-based assay format developed with human Arf1 as a model and reported here, as well as potentially allowing an assessment of its activation status in parasites using pull-down assays^37^.

Like its human counterpart, we confirmed that *Pf*Arf1 is susceptible to GDP/GTP nucleotide exchange stimulated by a Sec7 domain. Having used a human cytohesin domain for this purpose, we are currently exploring whether the nucleotide exchange activity extends to the predicted endogenous *P. falciparum* ArfGEF, despite the unusual secondary structure arrangement of its Sec7 domain^34,35^. In addition, we confirmed that *Pf*Arf1 deactivation can be achieved *in vitro* using the model GAP domain of human ArfGAP1 and that the GAP domains of the two putative *P. falciparum* ArfGAPs have equivalent catalytic GAP activities (based on EC_50_ values obtained in the assay format used here). Interestingly, despite the *Pf*Arf1 GAP activity displayed by the GAP domain of *Pf*ArfGAP2 and its presence in the parasite blood stages^43,44^, it has been reported as non-essential for blood-stage parasite survival, in contrast to *Pf*ArfGAP1, *Pf*Arf1 and the putative ArfGEF^39,40^. Along with the co-localisation of *Pf*Arf1 with the Golgi marker GRASP and the BFA sensitivity of parasite secretion and Golgi structure^27–32^, this may suggest that the latter trio of proteins form the regulatory network that mediates Arf GTPase-dependent trafficking of secretory proteins through the parasite Golgi apparatus. However, we recognise the caveat that we have performed the assays with truncated *Pf*Arf1 and *Pf*ArfGAP1 and that interaction *in vitro* does not necessarily translate into temporal and spatial co-recruitment and interaction on membrane surfaces *in vivo*. Potentially, this could be interrogated by parasite co-localisation experiments and assessing the effect of specific ArfGEF and ArfGAP1 inhibitors on *Pf*Arf1 activation status in parasites.

In addition to exploring the activity of *Pf*Arf1 regulatory proteins, the motivation for developing the assay described here was to establish an assay that can robustly detect the inhibition of the Arf1 activation/deactivation cycle and is amenable to screening compound libraries in a microtiter plate-based format. Conceptually, Arf function can be disrupted by inhibiting GTP binding, effector binding, GEF-mediated nucleotide exchange or GAP-mediated GTP hydrolysis. As opposed to inhibiting the binding of substrates/co-factors of traditional metabolic enzymes, protein-protein interactions are extremely challenging to interrupt with drug-like molecules^45,46^. It is therefore encouraging that this has been achieved with Arf1 (as well as Arf6^47^), with the application of developing potential anti-cancer agents in mind^10^. The focus of these studies has been on inhibitors of GEF-mediated Arf1 activation, but also includes the discovery of the GAP inhibitors QS11 and its derivatives^11,16–22^. To support inhibitor discovery, plate-based human Arf1 screening assays that have been reported include a FRET assay for GEF activity^48^, a fluorescence polarisation assay for GAP activity^49^, and an additional fluorescence polarisation aptamer displacement assay specific for cytohesins and used to identify SecinH3^20^. Relevant to these efforts, we show that the assay format reported here can competently detect the *in vitro* inhibition of ARNO Sec7-mediated human and *P. falciparum* ^NΔ17^Arf1 activation by SecinH3, as opposed to BFA and Golgicide A, as well as inhibition of the deactivation of both proteins by human ArfGAP1 using QS11. In addition, in a preliminary screen of a limited α-helix mimetic library, we identified Chem1099 as a low micromolar *in vitro* inhibitor of ^NΔ17^*Pf*Arf1 deactivation by the GAP domain of *Pf*ArfGAP1, further supporting the notion that ArfGAP activity can potentially be inhibited by small chemical compounds and, given the inactivity of Chem1099 against the GAP domains of *Hs*ArfGAP1 and *Pf*ArfGAP2, that this can be achieved selectively. In light of the reported essentiality of *Pf*Arf1 and *Pf*ArfGAP1 in blood-stage parasites, it is encouraging that Chem1099 inhibits blood-stage *P. falciparum*, albeit with a moderate IC_50_ of 14 µM compared to the low nanomolar activities obtained with standard antimalarials^50^. However, the assumption that parasite inhibition is due to GAP inhibition is a tenuous one in the absence of extensive mode of action or validation studies. Validation experiments could conceptually include an assessment of the effect of Chem1099 on parasite Golgi structure and function (e.g. through secretion assays), effect of Chem1099 on Arf1 activation status in parasites using pull-down assays on treated parasite lysates, and assessment of Chem1099 IC_50_ modulation in ArfGAP1 overexpressing or silenced transgenic parasite lines. We are currently expanding our screening of libraries for *Pf*ArfGAP1 inhibitors, coupled with biological assays to determine if this avenue of disrupting the *Pf*Arf1 activation cycle is detrimental to parasite viability.

## Methods

### Plasmid constructs and protein expression

For the *E. coli* expression of the GST-GGA3^GAT^ fusion protein (GST fused to the GAT domain - amino acids 107–286 - of human GGA3), pGEX-4T-2/hGGA3(GAT) (Addgene plasmid #79436, donated by Kazuhisa Nakayama) was used. The other coding sequences were ligated into the *Nhe*I/*Bam*HI (Arf1 sequences) or *Nhe*I/*Xho*I sites of pET-28a(+) for expression as His-tagged proteins. The coding sequence of human Arf1 minus the N-terminal 17 amino acids (^NΔ17^*Hs*Arf1) was PCR amplified from pARF1-CFP (Addgene plasmid #11381, donated by Joel Swanson) and the corresponding *P. falciparum* Arf1 sequence (^NΔ1^7*Pf*Arf1) from the full length *Pf*Arf1 sequence (PlasmoDB ID PF3D7_1020900) codon-optimised for human expression, synthesised and cloned into pBluescript II by GenScript (Hong Kong). The sequences for the GAP domain of human ArfGAP1 (*Hs*ArfGAP1^GAP^; amino acids 1–140; NCBI sequence NP_060679.1), Sec7 domain of ARNO (ARNO^Sec7^; amino acids 51–253; NP_004219.3) and the putative GAP domain of *P. falciparum* ArfGAP2 (*Pf*ArfGAP2^GAP^; amino acids 1–161; PF3D7_0526200.1) were codon optimised for *E. coli* expression and cloned into pET-28a by GenScript. The sequence encoding the putative GAP domain of *P. falciparum* ArfGAP1 (*Pf*ArfGAP1^GAP^; amino acids 1–161; PF3D7_1244600) was PCR amplified from *P. falciparum* strain 3D7 genomic DNA. T7 Express lysY *E. coli* (New England Biolabs) cultured in LB broth was used as expression host for all proteins. Expression was induced after bacterial density had reached OD_600_ 0.5–0.8 with 1 mM IPTG for 3 hours at 37 °C. Bacteria harvested from the induced cultures were lysed by a freeze/thaw cycle, resuspension in buffer containing 2 mg/mL lysozyme and probe sonication. Proteins were purified from the soluble supernatants by nickel-NTA agarose (His-tagged proteins) or glutathione agarose (GST-GGA3^GAT^) affinity chromatography. Purified proteins were buffer exchanged into assay buffer (25 mM HEPES, 150 mM KCl, 1 mM MgCl_2_, 1 mM DTT, pH 7.4) using desalting columns and protein concentrations determined using Bradford protein assay. Glycerol was added to a final concentration of 40% (v/v) and the proteins stored at −20 °C until use. More details on protein expression and purification are given in the  Supplementary Information (Fig. S2).

### Nucleotide loading of Arf1 proteins

To preload ^NΔ17^*Hs*Arf1 with GTP or GDP, the protein was diluted to a final concentration of 5 µM in assay buffer (25 mM HEPES, 150 mM KCl, 1 mM MgCl_2_, 1 mM DTT, pH 7.4) supplemented with 2 mM EDTA and 50 µM GTP or GDP and incubated at 25 °C for 60 minutes. MgCl_2_ was added to a final concentration of 3 mM and incubation continued for a further 10 min. Nucleotide loading of ^NΔ1^7*Pf*Arf1 was carried out in the same manner, except that 20 mM EDTA and 30 mM MgCl_2_ was used. To monitor nucleotide binding, intrinsic tryptophan fluorescence was measured at Ex_297_/Em_340_ in a Spectramax M3 plate reader (Molecular Devices). In addition, after completion of nucleotide loading, proteins were analysed in a gel shift (native PAGE) assay. Native PAGE was carried out with a 12% resolving gel and 4% stacking gel using normal SDS-PAGE conditions, except that SDS was omitted from all buffers and reducing agents were omitted from the sample buffer. After electrophoresis, the gel was stained with Coomassie Brilliant Blue.

### Plate-based GST-GGA3^GAT^ binding assay

His-tagged ^NΔ17^*Hs*Arf1 or ^NΔ1^7*Pf*Arf1 preloaded with GTP or GDP were diluted to 1 µM in assay buffer supplemented with 1% (w/v) bovine serum albumin (BSA), transferred to a Ni-NTA HisSorb 96-well plate (Qiagen) (50 µL per well) and incubated at 4 °C for 30 min with gentle agitation. GST-GGA3^GAT^ in 50 µL assay buffer was added to a final concentration of 1 µM and incubation continued for an additional 60 min at 4 °C. The protein solutions were aspirated, the wells washed twice in assay buffer containing 0.1% (v/v) Tween-20 followed by four additional washes in assay buffer. GST assay buffer (2 mM reduced L-glutathione and 1 mM 1-chloro-2,4-dinitrobenzene in phosphate-buffered saline, pH 7.4), pre-equilibrated to room temperature, was added to each well (200 µL/well), the plate incubated at room temperature for 30 min and absorbance read at 340 nm in a Spectramax M3 plate reader. Background absorbance readings were obtained from triplicate wells incubated with GST-GGA3^GAT^ in the absence of immobilised Arf1 and the mean absorbance subtracted from the absorbance values of the experimental GST-GGA3^GAT^ wells. Plates were prepared for re-use by rinsing the plate wells in water followed by a 10 min incubation in stripping buffer (20 mM sodium phosphate, 500 mM NaCl, 50 mM EDTA, pH 7.4), an additional wash in water and a 10 min incubation in recharging solution (0.1 M NiSO_4_). After a final rinse in water, the plates were used immediately.

### ARNO-mediated nucleotide exchange and GAP-mediated GTP hydrolysis assays

For nucleotide exchange assays, 1 μM ^NΔ17^*Hs*Arf1 or ^NΔ1^7*Pf*Arf1 preloaded with GDP was incubated with 0.2 µM ARNO^Sec7^ and 50 μM GTP in assay buffer containing 1% BSA in round-bottom plates (50 µL per well) at 37 °C for 30 minutes with continuous agitation. The reactions were transferred to a Ni-NTA plate and the plate-based GST-GGA3^GAT^ binding assay continued as described above. Negative controls included reactions without ARNO, without GTP, or without either. GAP assays were carried out in the same manner, except that Arf1 proteins preloaded with GTP were used, ARNO was replaced with 0.1 µM of the relevant GAP domain (*Hs*ArfGAP1^GAP^, *Pf*ArfGAP1^GAP^, *Pf*ArfGAP2^GAP^) and the addition of GTP was omitted. Negative controls consisted of reactions lacking the GAP domains. To assess the inhibition of nucleotide exchange or GTP hydrolysis, 10 mM stocks of brefeldin A (BFA; Sigma-Aldrich), Golgicide A (GA; Sigma-Aldrich), SecinH3 (Tocris Bioscience) and QS11 (Tocris Bioscience) were prepared in DMSO. The inhibitors were added to the reactions in the round-bottom plate wells to a final concentration of 50 µM [inhibitors were added to the Arf1 solutions immediately before adding ARNO (BFA, GA or SecinH3) or the GAP domains (QS11)]. A corresponding volume of DMSO was added to control reactions lacking the inhibitors (solvent vehicle controls). GAP titration experiments with ^NΔ1^^7^*Pf*Arf1 were carried out as described above, except that incubations were carried out with 1 µM ^NΔ1^7*Pf*Arf1-GTP and 3-fold serial dilutions (0.5–0.002 µM) of the GAP domains. For compound library screening, 50 µL assay buffer containing 1% BSA, 1 µM ^NΔ1^7*Pf*Arf1-GTP and 0.1 µM *Pf*ArfGAP1^GAP^ was incubated in the presence of 50 µM of the test compounds in round-bottom plates for 30 minutes at 37 °C (compounds were added to the reaction mixture before the addition of the GAP domain). The reaction mixtures were transferred to Ni-NTA plates and the GST-GGA3^GAT^ binding assay continued as described above. Dose-dependent inhibition of *Pf*ArfGAP1^GAP^ by Chem1099 was determined in the same manner, using 3-fold serial dilutions of the compound. Percentage inhibition of GAP activity at the respective compound concentrations was calculated from Abs_340_ readings relative to those obtained with ^NΔ1^7*Pf*Arf1-GTP incubated with *Pf*ArfGAP1^GAP^ without Chem1099 (0%) and ^NΔ1^7*Pf*Arf1-GTP incubated without *Pf*ArfGAP1^GAP^ (100%). A dose-response curve of percentage inhibition vs. Log[Chem1099] was generated and the IC_50_ determined using non-linear regression analysis with GraphPad Prism (v.8.2.0).

### Antiplasmodial assay

This was carried out as described previously^51^. Briefly, cultures of *Plasmodium falciparum* (3D7) parasites in a 96-well plate were incubated with a 3-fold serial dilution of Chem1099 (100–0.046 µM) for 48 h and parasite levels assessed using a colorimetric plasmodial lactate dehydrogenase (pLDH) assay^52^. Absorbance readings were converted to percentage parasite viability relative to readings obtained from control wells (parasite cultures without Chem1099) and IC_50_ derived by non-linear regression analysis of the resulting % viability vs. Log(Chem1099 concentration) using GraphPad Prism.

## Supplementary information

Supplementary Information.

## Data Availability

The majority of the data generated or analysed during this study are included in this article and Supplementary Information. Data not shown are available by request from the corresponding author.
