# Supplementary material for: Detection of the in vitro modulation of Plasmodium falciparum Arf1 by Sec7 and ArfGAP domains using a colorimetric plate-based assay
Source: Sci Rep. 2020 Mar 6;10:4193. doi: 10.1038/s41598-020-61101-3 (PMC7061341; doi:10.1038/s41598-020-61101-3)
Supplement: Supplementary file 1 — Supplementary Information. [file 41598_2020_61101_MOESM1_ESM.pdf]

SUPPLEMENTARY INFORMATION

Detection of the *in vitro* modulation of *Plasmodium falciparum* Arf1 by Sec7 and ArfGAP domains using a colorimetric plate-based assay.

Tarryn Swart, Farrah D. Khan, Apelele Ntlantsana, Dustin Laming, Clinton G. L. Veale, Jude M. Przyborski, Adrienne L. Edkins, Heinrich C. Hoppe

1. Principle of the colorimetric plate-based GST-GGA3 binding assay.

The principle of the assay is diagrammatically illustrated in Fig. S1.
Step 1: GDP-preloaded His-tagged $^{N\Delta17}$Arf1 is incubated with GTP and the Sec7 domain of ARNO, stimulating GDP/GTP exchange by the Arf1 protein. Alternatively, GTP-preloaded His-tagged $^{N\Delta17}$Arf1 is incubated with a GAP domain, stimulating GTP hydrolysis by the Arf1 protein.
Step 2: The reaction mixtures are transferred to a Ni-NTA 96-well plate, allowing the His-tagged $^{N\Delta17}$Arf1 protein to bind to the nickel-coated well surface.
Step 3: The GAT domain of GGA3, fused to GST, is added to the plate, allowing the GAT domain to bind selectively to $^{N\Delta17}$Arf1-GTP.
Step 4: Unbound proteins are washed off, followed by the addition of GST enzyme substrate consisting of 1-chloro-2,4-dinitrobenzene (CDNB) and reduced glutathione (GSH). Formation of the reaction product – glutathione conjugated to CDNB (GS-CDNB) – is measured by absorbance at 340 nm.

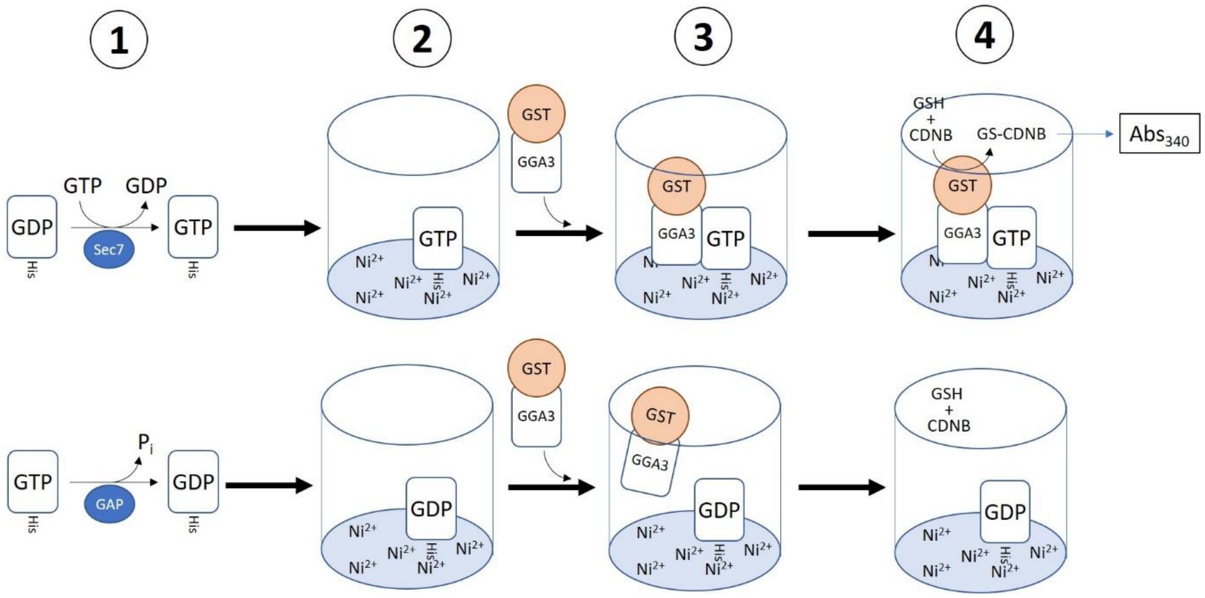

Figure S1. Principle of the colorimetric plate-based GST-GGA3 binding assay.

2. Expression and purification of recombinant proteins.

T7 Express lysY *E. coli* (New England Biolabs) transformed with the expression constructs were cultured at 37°C in 250 mL LB broth containing 50 µg/mL kanamycin or ampicillin. When bacterial density had achieved an OD_{600} reading of 0.5 – 0.8, isopropyl β-D-1-thiogalactopyranoside (IPTG) was added to a final concentration of 1 mM and incubation continued for 3 hours at 37°C. The bacteria were pelleted by centrifugation, washed in equilibration buffer (50 mM Tris-HCl, 20 mM imidazole, pH 8.0 for His-tagged $^{N\Delta17}$Arf1, GAP domains and ARNO^{Sec7} domain; 40 mM Tris-HCl, 150 mM NaCl, 1mM phenylmethylsulfonyl fluoride, 0.2 % (v/v) Triton X-100, pH 8.0, for GST-GGA3^{GAT}) and stored frozen at -80°C. After thawing, the bacterial pellets were resuspended in equilibration buffer containing 2 mg/mL lysozyme, incubated for 30 min on ice and sonicated for two cycles of 1 min each at 60 Hz using a probe sonicator. Insoluble material was removed by centrifugation at 14000 *g* for 30 min and sequential filtration of the supernatants through 0.45 µm and 0.2 µm filters. The samples were applied to Ni-NTA agarose or glutathione agarose columns, the columns washed in equilibration buffer and the proteins eluted with 3 mL 50 mM Tris-HCl, 500 mM imidazole, pH 8.0 (Ni-NTA columns) or 50 mM Tris-HCl, 10 mM reduced L-glutathione, pH 9.5 (glutathione agarose columns). For buffer exchange, the eluates were applied to PD-10 desalting columns (GE Healthcare) pre-equilibrated with assay buffer (25 mM HEPES, 150 mM KCl, 1 mM $MgCl_2$, 1 mM DTT, pH 7.4) and the proteins eluted with 3.5 mL assay buffer. Protein concentration was determined with Bradford reagent (Sigma-Aldrich) using bovine serum albumin serial dilutions as a reference standard, glycerol was added to the protein samples to a final concentration of 40% (v/v) and the samples stored at -20°C.

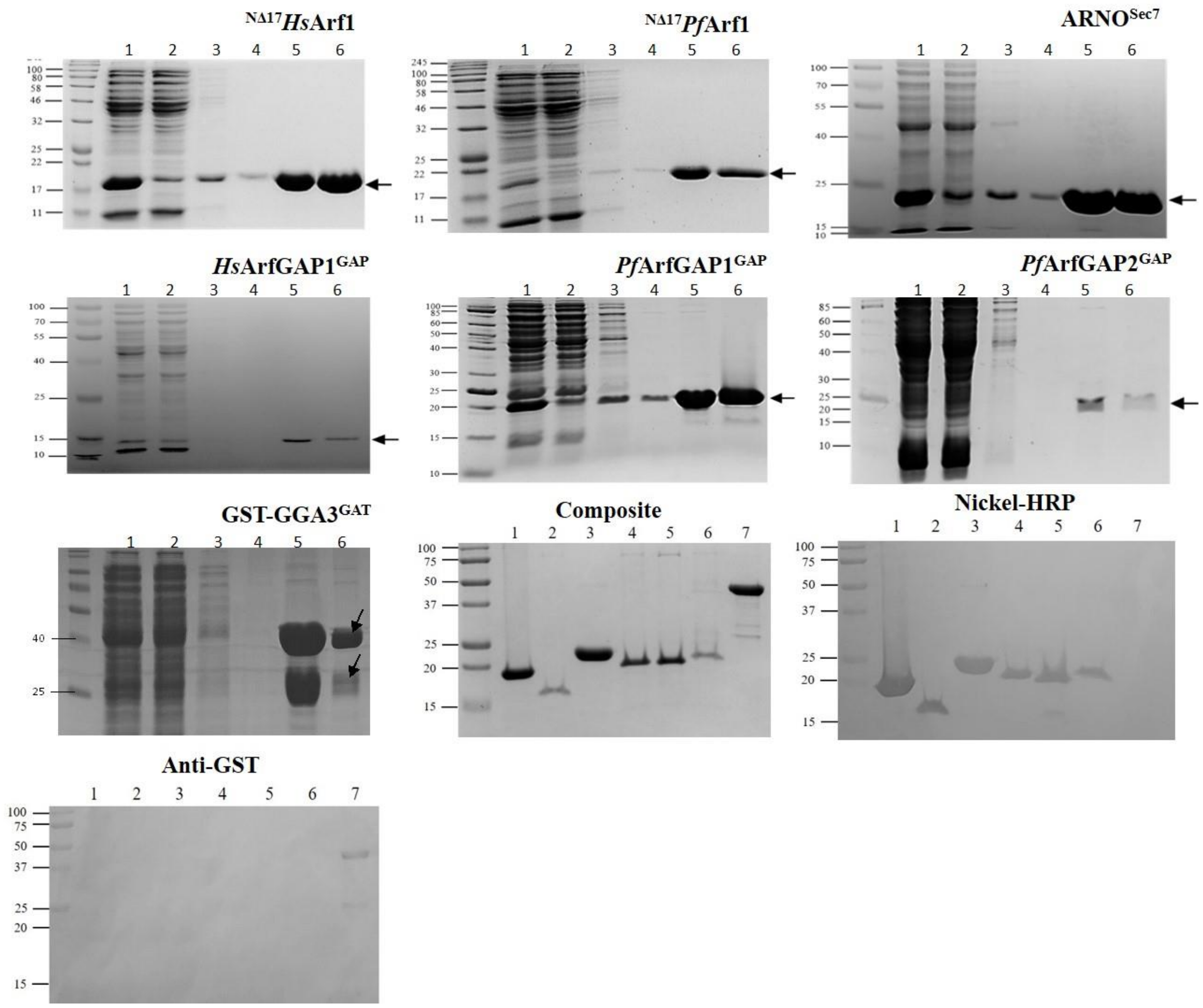

Figure S2. Purification of recombinant proteins used in this study. Purification samples were analysed by SDS-PAGE and stained with Coomassie. Lane 1: Soluble bacterial supernatant. Lane 2: Column flow-through. Lanes 3-4: Sequential column washes. Lane 5: Eluted protein. Lane 6: Final desalted protein sample. Arrows indicate the position of the position of purified proteins. The lower arrow in the GST-GGA3^{GAT} gel indicates co-purified free GST. The composite gel contains all the purified proteins used in this study, stained with Coomassie. The purified proteins were additionally probed by western blotting with a 1:5000 dilution of HisDetector nickel-HRP (SeraCare) to detect His-tagged proteins, or with a 1:1000 dilution of rabbit anti-GST (Sigma-Aldrich) and peroxidase conjugated goat anti-rabbit Ig (SeraCare) to detect GST-tagged proteins. Bands were detected by incubating the blots in TMB membrane peroxidase substrate (SeraCare). The lanes in the composite Coomassie stained gel and corresponding blots (nickel-HRP, anti-GST) contain the following: 1: $^{N\Delta17}$*Hs*Arf1; 2: *Hs*ArfGAP1^{GAP}; 3: ARNO^{Sec7}; 4: $^{N\Delta17}$*Pf*Arf1; 5: *Pf*ArfGAP1^{GAP}; 6: *Pf*ArfGAP2^{GAP}; 7: GST-GGA3^{GAT}.

Expected molecular weights of purified proteins (incl. the N-terminal His-tag extension encoded by the pET-28a vector) were:
$^{N\Delta17}$*Hs*Arf1: 21.1 kDa, $^{N\Delta17}$*Pf*Arf1: 21.1 kDa, ARNO^{Sec7}: 25.6 kDa, *Hs*ArfGAP1^{GAP}: 18.5 kDa, *Pf*ArfGAP1^{GAP}: 20.7 kDa, *Pf*ArfGAP2^{GAP}: 20.5 kDa, GST-GGA3^{GAT}: 46.4 kDa.

3. Colorimetric GST-GGA3 binding assay with free GST; GGA3 co-precipitation assay.

As is common with GST fusion proteins, the GST-GGA3^{GAT} recombinant protein preparation occasionally includes free GST (Fig. S2). This leads to an overestimation of the GST-GGA3^{GAT} concentration (determined by Bradford assay) used in the Arf1 binding assays. To determine if the free GST may be responsible for the binding signal obtained when GST-GGA3^{GAT} preparations are incubated with GTP-loaded Arf1, the colorimetric plate-based binding assay was repeated with immobilised $^{N\Delta17}$*Pf*Arf1-GTP and -GDP incubated respectively with GST-GGA3^{GAT} and untagged GST obtained by expression and purification from *E. coli* transformed with empty pGEX-4T-2 vector. As shown in Fig. S3a, unlike GST-GGA3^{GAT}, the free GST failed to bind to or discriminate between GDP- and GTP-loaded $^{N\Delta17}$*Pf*Arf1 and produced binding signals equivalent to those obtained in wells incubated with GST alone. This also suggests that binding of $^{N\Delta17}$*Pf*Arf1-GTP to the GST-GGA3^{GAT} fusion protein is due to recognition of the GGA3^{GAT} portion of the protein, not the GST fusion partner.

To further confirm binding of GTP-bound $^{N\Delta17}$*Pf*Arf1 to GGA3, a bead co-precipitation assay was performed (Fig. S3b, c). $^{N\Delta17}$*Pf*Arf1-GTP and -GDP (5 µM in assay buffer containing 1% BSA and 0.1% Tween-20) were respectively incubated with 5 µL beads coated with GGA3 protein binding domain (Cell Biolabs) at 4°C for 60 min. The beads were separated from the supernatant and washed using brief centrifugations in 0.45 µM Spin-X centrifuge tube filters (Corning) and resuspended in SDS-PAGE sample buffer. The bead pellet sample along with samples of the supernatant and wash were analysed on SDS-PAGE gels stained with Coomassie (Fig. S3b) or probed with anti-Arf1 antibodies (Fig. S3c). For the latter, the SDS-PAGE gel was transblotted onto a nitrocellulose membrane and incubated with a 1:1000 dilution of monoclonal anti-Arf1 antibody 1D9 (Novus Biologicals), followed by incubations with peroxidase-conjugated goat anti-mouse Ig and TMB peroxidase substrate. In both the Coomassie-stained gel and western blot, $^{N\Delta17}$*Pf*Arf1-GTP was absent from the supernatant after bead incubation and exclusively present in the bead pellet, while the opposite was the case with $^{N\Delta17}$*Pf*Arf1-GDP. This confirms selective binding of GTP- vs. GDP-bound $^{N\Delta17}$*Pf*Arf1 to GGA3.

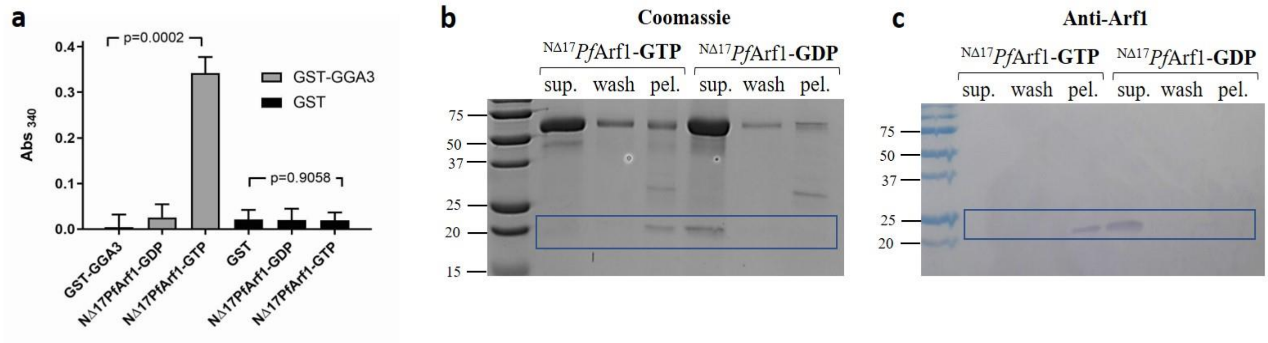

Figure S3. Selective binding of $^{N\Delta17}$*Pf*Arf1-GTP to GGA3. **a.** $^{N\Delta17}$*Pf*Arf1-GTP and -GDP (1 µM) were immobilised on Ni-NTA coated plates by incubation for 30 min at 4°C. GST-GGA3^{GAT} or GST were added to the wells to a final concentration of 1 µM and incubation

continued for 60 min. After washing, bound GST was detected at 340 nm by adding GST substrate solution and incubating for 30 min. Controls consisted of wells incubated with GST-GGA3^{GAT} or GST in the absence of immobilised $^{N\Delta17}$*Pf*Arf1. Incubations were conducted in technical triplicate and error bars indicate standard deviation. P-values were calculated by two-tailed t-tests. **b, c.** GGA3-coated beads were incubated with $^{N\Delta17}$*Pf*Arf1-GTP and -GDP and the presence of $^{N\Delta17}$*Pf*Arf1 in the supernatant (sup.), bead wash and bead pellet (pel.) determined by analysing the respective fractions on a Coomassie-stained SDS-PAGE gel (**b**) or by western botting with 1D9 anti-Arf1 monoclonal antibody (**c**).

4. GAP domains used in this study.

GAP domain sequences used in this study were amino acids 1-140 of human ArfGAP1 (NCBI sequence NP_060679.1), amino acids 1-161 of *P. falciparum* ArfGAP1 (PlasmoDB entry PF3D7_1244600) and amino acids 1-161 of *P. falciparum* ArfGAP2 (PlasmoDB entry PF3D7_0526200.1). Alignments of the full sequences used are shown in Fig. S3, highlighting the catalytic arginine residue required for GAP activity[1] (arrow).

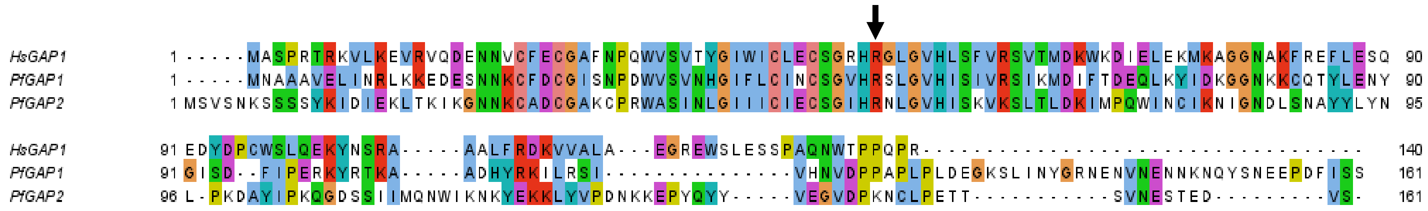

Figure S4. The GAP domain sequences used in this study. Alignment was carried out using Clustal Omega[2] and the figure prepared with Jalview[3]. The conserved catalytic R residue is indicated by the arrow.

5. Compound library screening.

A library of 1120 BioFocus α-helix mimetics were screened for their ability to inhibit the *Pf*ArfGAP1^{GAP}-mediated stimulation of GTP hydrolysis by *Pf*Arf1. GAP reactions were carried out in round-bottom 96-well plates and consisted of 1 µM $^{N\Delta1}$7*Pf*Arf1-GTP, 0.1 µM *Pf*ArfGAP1^{GAP} and 50 µM test compound incubated in a total volume of 100 µl assay buffer (25 mM HEPES, 150 mM KCl, 1 mM $MgCl_2$, 1 mM DTT, pH 7.4) for 30 min at 37°C. After the reaction, the mixtures were transferred to 96-well nickel-NTA plates and the colorimetric GST-GGA3^{GAT} binding assay carried out as described in the main text. Each plate contained wells with negative ($^{N\Delta1}$7*Pf*Arf1-GTP and *Pf*ArfGAP1^{GAP} without compound, representing 0% inhibition) and positive ($^{N\Delta1}$7*Pf*Arf1-GTP alone, representing 100% inhibition) control reactions, and % inhibition by individual compounds was calculated from the respective Abs_{340} values obtained for their reactions relative to the mean values obtained for the controls. Hits were defined as compounds inhibiting the GAP reaction by ≥ 70% and yielded 13 compounds (Fig. S4). Of these, 5 failed to reproduce ≥ 70% inhibition in a follow-up confirmatory screen. The remaining 8 compounds were subjected to dose-response evaluation using 3-fold serial

dilutions of the compounds (50 – 0.2 µM) from which Chem1099 emerged as the most active compound (IC_{50} = 4.7 µM; graph shown in main text – Fig. 5e).

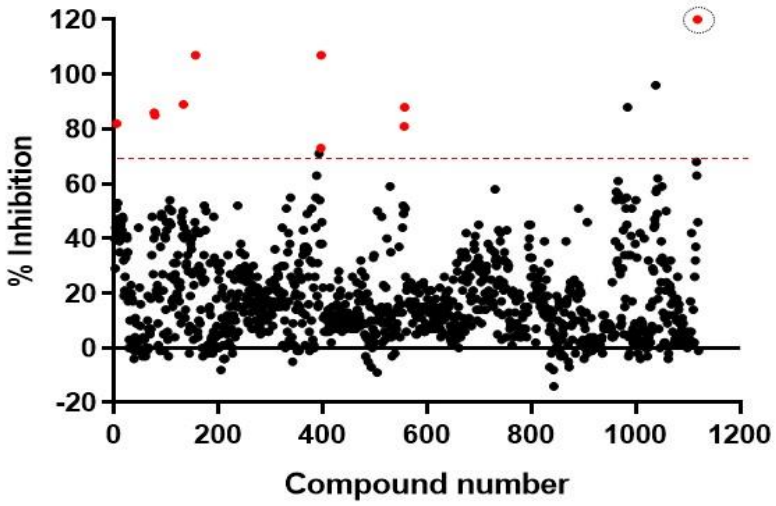

Figure S5. Primary screening of a BioFocus α-helix mimetic library for inhibition of *Pf*ArfGAP1^{GAP}-mediated deactivation of $^{N\Delta 1}$7*Pf*Arf1-GTP. Hits were defined as compounds yielding ≥ 70% inhibition of the reaction (indicated by the red dotted line). Red dots represent hit compounds that reproduced this level of activity in a second confirmation assay. The circled dot represents Chem1099.

6. Native PAGE

To monitor Arf1 conformational changes due to nucleotide (GTP vs. GDP binding), native PAGE was performed on samples of GTP- and GDP-preloaded $^{N\Delta 17}$Arf1 proteins (Fig. S6).

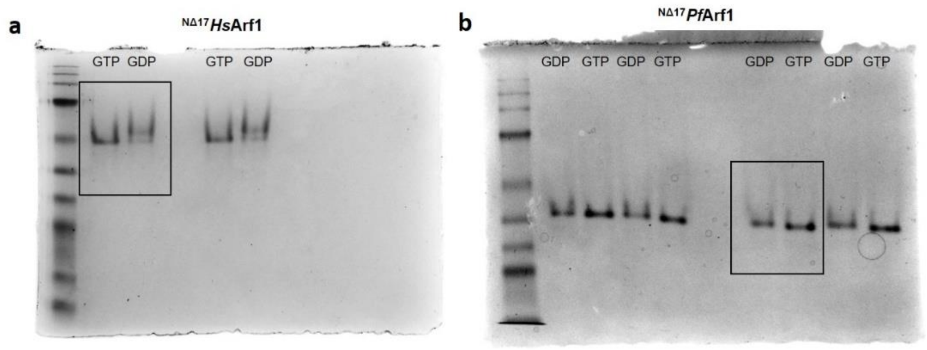

Figure S6. Native PAGE analysis of GTP- and GDP-preloaded Arf1 proteins. Samples of $^{N\Delta 1}$7*Hs*Arf1-GTP and -GDP (**a**) or $^{N\Delta 1}$7*Pf*Arf1-GTP and -GDP (**b**) were loaded in alternating wells and electrophoresed in a 12% non-denaturing PAGE gel that was stained with Coomassie. The left-hand lane contains a pre-stained SDS-PAGE molecular weight marker that was included to track electrophoresis progress, not for mw determination. Black rectangles indicate cropped regions of the respective gels used to compile Fig. 1.

References.

1. Kahn, R.A. *et al.* Consensus nomenclature for the human ArfGAP domain-containing proteins. *J. Cell Biol*. **182**, 1039-1044 (2008).
2. Madeira, F. *et al.* The EMBL-EBI search and sequence analysis tools APIs in 2019. *Nucl. Acids Res*. **47**, W636-W641 (2009)
3. Waterhouse, A. M., Procter, J. B., Martin, D. M. A., Clamp, M. & Barton, G. J. Jalview Version 2 – a multiple sequence alignment editor and analysis workbench. *Bioinformatics* **25**, 1189-1191 (2009).
